# Automatic detection method for tobacco beetles combining multi-scale global residual feature pyramid network and dual-path deformable attention

**DOI:** 10.1038/s41598-024-55347-4

**Published:** 2024-02-28

**Authors:** Yuling Chen, Xiaoxia Li, Nianzu Lv, Zhenxiang He, Bin Wu

**Affiliations:** 1 School of Information Engineering, Southwest University of Science and Technology, Mianyang, 621010 Sichuan China; 2 Robot Technology Used for Special Environment Key Laboratory of Sichuan Province, Mianyang, 621010 Sichuan China; 3 Xinjiang Institute of Technology, Aksu, 13558 Xinjiang China; 4 Mianyang Teachers’ College, Mianyang, 621000 Sichuan China; 5 Tianfu College of Southwest University of Finance and Economics, Mianyang, 621000 Sichuan China

**Keywords:** Tobacco beetle, Small object, Tobacco beetle dataset, Deformable convolutional, Attention mechanism, Feature pyramid network, Engineering, Mathematics and computing, Information technology

## Abstract

Aiming at the problems of identifying storage pest tobacco pest beetles from images that have few object pixels and considerable image noise, and therefore suffer from lack of information and identifiable features, this paper proposes an automatic monitoring method of tobacco beetle based on Multi-scale Global residual Feature Pyramid Network and Dual-path Deformable Attention (MGrFPN-DDrGAM). Firstly, a Multi-scale Global residual Feature Pyramid Network (MGrFPN) is constructed to obtain rich high-level semantic features and more complete information on low-level features to reduce missed detection; Then, a Dual-path Deformable receptive field Guided Attention Module (DDrGAM) is designed to establish long-range channel dependence, guide the effective fusion of features and improve the localization accuracy of tobacco beetles by fitting the spatial geometric deformation features of and capturing the spatial information of feature maps with different scales to enrich the feature information in the channel and spatial. Finally, to simulate a real scene, a multi-scene tobacco beetle dataset is created. The dataset includes 28,080 images and manually labeled tobacco beetle objects. The experimental results show that under the framework of the Faster R-CNN algorithm, the detection precision and recall rate of this method can reach 91.4% and 98.4% when the intersection ratio (IoU) is 0.5. Compared with Faster R-CNN and FPN, when the intersection ratio (IoU) is 0.7, the detection precision is improved by 32.9% and 6.9%, respectively. The proposed method is superior to the current mainstream methods.

## Introduction

The tobacco beetle is a worldwide storage pest. Their bodies and dung pollute tobacco leaves and products, seriously endangering the availability and quality of tobacco leaves. With the continuous expansion of the production scale of the cigarette industry around the world, the storage scale of tobacco leaves, the most important raw material of cigarettes, has also expanded, and due to the emergence of new tobacco technology, tobacco leaves generally need to be alcoholized in the warehouse for 2 to 3 years, which will lead to the expansion of the scope and scale of tobacco a hazard. The annual loss of tobacco leaves due to tobacco beetle accounts for 1% to 5% of the total number of stored tobacco leaves worldwide, and the loss caused by tobacco beetle accounts for more than 98% of the total loss. In the process of tobacco beetle control, pest monitoring is the most basic and important part of tobacco beetle control. The traditional detection method for tobacco beetles is mainly to arrange tobacco beetle traps at monitoring points in the tobacco-making workshop, and then manually count the number of tobacco beetles on each trap regularly. However, the manual counting method has the disadvantages of high time cost, low monitoring efficiency, poor real-time performance, inaccurate data, and so on. Therefore, it is of great significance for cigarette production quality control by using computer vision technology to realize automatic image acquisition and tobacco beetle detection.

Recently, there are many research results on image processing and analysis technology in crop pest detection and recognition^1^, but the research on storage is less. Typical image analysis techniques for storage pest monitoring focus on pest classification^2^ rather than pest detection (location and recognition). The purpose of detection is to obtain the situation of tobacco beetles by locating and recognizing each pest instance from trapping points image data. Then, through the analysis of historical data to guide the selection of monitoring points and accurate pest control. It plays an important role in the scientific and effective monitoring and control of storage pests. Therefore, for the monitoring of storage pests, it is necessary to establish a new automatic monitoring method by deeply mining more valuable information as the recognition features of pest detection, and establish an intelligent and efficient storage pest monitoring system. In this context, this paper aims to solve the problems existing in the current warehouse pest detection tasks by studying advanced deep learning methods and finding a practical and effective pest monitoring solution.

The major contributions of this paper are as follows: (1) A CNN (Convolutional Neural Networks, CNN) based two-stage tobacco beetle detection method combining Multi-scale Global residual Feature Pyramid Network and dual-path deformable attention (MGrFPN-DDrGAM) are designed; (2) Two new modules are designed: Multi-scale Global Residual Feature Pyramid (MGrFPN) and Dual-path Deformable receptive field Guided Attention Module (DDrGAM), which are used for the acquisition of rich multi-scale features an automatic fusion of effective features, respectively; (3) A small object detection dataset of tobacco beetles in the complex environment was established to simulate the detection of real stored tobacco beetles, including 28,080 images under various conditions, such as whether it is dense, whether there is shredded tobacco, and whether the illumination is uniform. The experimental results show that, under the framework of Faster R-CNN^3^, our approach MGrFPN-DDrGAM achieves over AP and Recall of 90.4% and 96.4% when the IoU is 0.5, which outperforms the current mainstream methods under different IoUs.

## Related work

Object detection mainly solves the problem of object localization and recognition in images or videos. At present, the mainstream object detection methods are mainly divided into two-stage and single-stage detection methods. The region-based two-stage detection algorithm is represented by the R-CNN^4^. The regression-based one-stage detection algorithms are represented by YOLO^5–7^ and SSD^8,9^. The two-stage algorithm has more advantages in detection and location accuracy, while the single-stage algorithm has more advantages in running efficiency. In recent years, Transformers have been widely used in the field of computer vision^10^, particularly demonstrating excellent performance in multiple tasks of salient object detection. Its advantage lies in the ability to capture global and local contextual information, without being limited by the fixed receptive field size in traditional methods. In addition, Transformers can also undergo end-to-end training to better learn salient features in images.

However, small objects occupy a small proportion of pixels in the image and have the features such as small coverage area and lack of information. Conventional object detection methods are used to detect small objects. The main difficulty is that while obtaining high-level semantic information, it will lose low-level detail information, which leads to missed detection of small objects. Small object detection has always been a difficult problem in the field of object detection. The general effect of using classical detection methods to detect small objects is not ideal. Judging from the detection results on PASCAL VOC^11,12^, MS-COCO^12^, and other datasets, they have poor detection results on small objects such as birds and ships but good on complex but larger objects. This shows that on the one hand, the feature network cannot learn specific features and express features, and on the other hand, the small object features extracted by the network can provide less information to the model. Therefore, Feature Pyramid Network (FPN)^13^ proposes a network structure that utilizes the inherent multi-scale pyramid structure of deep convolutional neural networks to construct feature pyramids. It takes into account features with different sizes, resolutions, and receptive fields by introducing a top-down approach with lateral connections to detect objects of different scales. To further improve the resolution of feature maps while maintaining the receptive field, DetNet^14^ employs dilated convolutionals and adds an extra stage. CARAFE^15^ uses a new upsampling method to replace the traditional upsampling method and integrates it into the feature pyramid to obtain a larger receptive field. ASPP^16^ employs dilated convolutionals with different dilation rates to expand the receptive field, which is applied to the input feature map in parallel to capture the contextual information of the image at multiple scales. To strengthen the fusion of information, RetinaNet^17^ is different from FPN in that it starts from the third-to-last layer for feature fusion. PANet^18^ adopts bottom-up secondary fusion to enhance the top-down FPN path, shorten the information transmission path, and make better use of the precise location information of low-level features. ASFF^19^ fuses the feature information of each layer feature information and adopts an attention mechanism to control different layers’ contributions. NAS_FPN^20^ aims to use neural architecture search to automatically learn a better feature pyramid network architecture for object detection. Then, BiFPN^21^ is artificially designed based on a neural architecture search to obtain a better feature pyramid network. DetectoRS^22^ adds additional feedback from FPN to the bottom-up backbone layer. PRB-FPN^23^ proposes a new parallel FP structure that employs bidirectional (top-down and bottom-up) fusion to maintain high-quality features for precise localization. A^2-FPN^24^ proposes a feature pyramid network based on attention aggregation to improve multi-scale feature learning through attention-guided feature aggregation.

**Figure 1:**
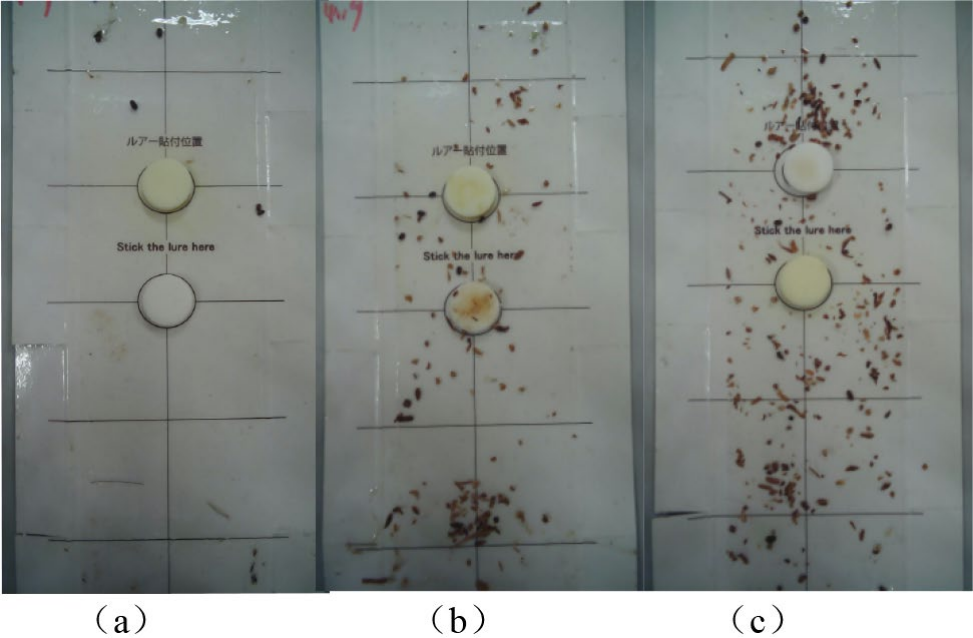
Typical examples of tobacco beetle data. (**a**) The bright and clean image, (**b**) the bright with little cut tobacco image, and (**c**) the bright with much cut tobacco image.

The above networks do not fully consider the effective fusion of information while increasing the receptive field, and most networks continue to add paths after obtaining high- and low-level fusion information, which improves the performance of the network at the cost of increasing network complexity. Without expanding the receptive field of the high-level network, the semantic information of the high-level feature map is still insufficient; In addition, the high-level features that have undergone multiple downsampling generally ignore more detailed information, resulting in extremely poor detection of small objects. Take the detection of the storage pest tobacco beetle as an example, as shown in Fig. 1 and Table 1. Due to many limiting factors, general deep learning methods are difficult to directly apply to tobacco beetle detection: (1) Tobacco beetle objects are much smaller than other small objects currently published and representative datasets, such as MS COCO^12^, small objects in public datasets such as Wider Face^25^ and City Persons^26^, tobacco beetles have lower resolution, carry less information, and are insensitive to features after image translation and rotation, resulting in weak feature expression ability. (2) The intuitive features such as texture, shape, or color of the tobacco beetle are easy to be confused with the background information (tobacco, dust, etc.), especially in an environment of uneven illumination, which leads to the sharp decline of image signal-to-noise ratio and object significance, therefore increases the likelihood of false detection. (3) The uneven distribution of size, posture, and density of tobacco beetles, and the diversity of collected image data, make detection more difficult. To solve the above problems, this paper aims to solve the problem of tobacco beetle detection by studying effective feature enhancement and feature fusion methods.

**Table 1:** Comparison of mall objects in different datasets.

Data set	Absolute size	Relative size	Aspect ratio
Tobacco beetle (ours)	46.9 ± 30.5	0.033 ± 0.021	1.471 ± 1.355
MS COCO^12^	99.5 ± 107.5	0.190 ± 0.203	1.214 ± 1.339
Wider face^25^	32.8 ± 52.7	0.036 ± 0.052	0.801 ± 0.168
City persons^26^	79.8 ± 67.5	0.055 ± 0.046	0.410 ± 0.008

## Materials and methods

### Combining multi-scale global residual feature pyramid network and dual-path deformable receptive field guided attention module methods

Tobacco beetles are very small, and their features are very weak, such as insufficient brightness and lack of edge information, so it is difficult to extract discriminating features. Furthermore, as shown in Fig. 1, the intuitive features of tobacco beetles, such as texture, shape, or color, can easily be confused with background information (such as tobacco, dust, etc.), leading to a complex background in images of tobacco beetles and consequently, a higher likelihood of false detections.

**Figure 2:**
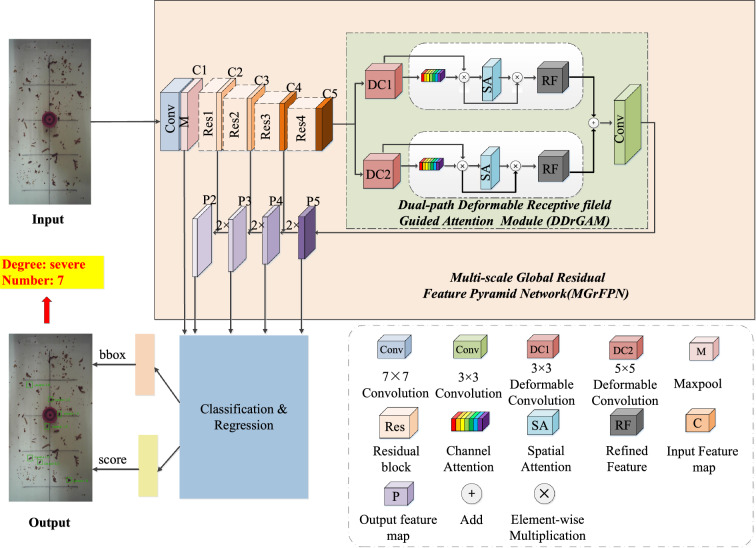
MGrFPN–DDrGAM network structure.

To improve the detection accuracy, according to the different sizes, postures, perspective changes, and other geometric deformations, distribution, and complex backgrounds of tobacco beetles in the images, we propose a Multi-scale Global residual Feature Pyramid Network and Dual-path Deformable Attention (MGrFPN-DDrGAM) method for tobacco beetles detection in complex environments. The method mainly consists of four parts: feature extraction network-Resnet50^27^, Multi-scale Global residual Feature Pyramid Network (MGrFPN), Dual-path Deformable receptive field Guided Attention Module (DDrGAM), and Object detection head of Faster R-CNN^3^ the network structure is shown in Fig. 2.

This structure of MGrFPN adopts multi-scale feature fusion, it makes full use of the shallow detail information and deep semantic information that are beneficial to the feature extraction of small objects. MGrFPN facilitates the differentiation between objects and background by analyzing characteristics such as the shape, size, color, and texture of the object, as well as the contextual information surrounding the object. To enhance the model's capability in scenarios where the background closely resembles the object, we introduce the DDrGAM. This module initiates with a regular sampling of the input feature map, followed by the application of deformable convolution kernels on the sampled features of the tobacco beetle to perform a weighted summation for feature aggregation. This approach adaptively selects the spatial domain of the tobacco beetle regions, thereby expanding the effective receptive field of the convolutional neural network. Subsequently, we have developed a feature attention block that assigns differential weights to various features. Through this mechanism, the network focuses more on relevant information while disregarding the irrelevant.

In the detection of tobacco beetles, acquiring detailed feature information of the beetles is critical for accurate localization and recognition of key points. Therefore, we have incorporated a spatial attention module that allocates greater weight to the spatial location information of the tobacco beetles within the acquired feature maps, thereby directing the network model to focus more on regions with useful information. This enables the model to concentrate more on the object areas while ignoring parts that resemble the background. Given that the object and background may exhibit similarities across different scales, we employ a multi-scale detection approach, conducting object detection across various scales to identify the most appropriate scale. This method reduces false detections caused by similarities between the object and the background, effectively enhancing the performance of small object detection, which can effectively improve the performance of small object detection.

### MGrFPN detection architecture

**Figure 3:**
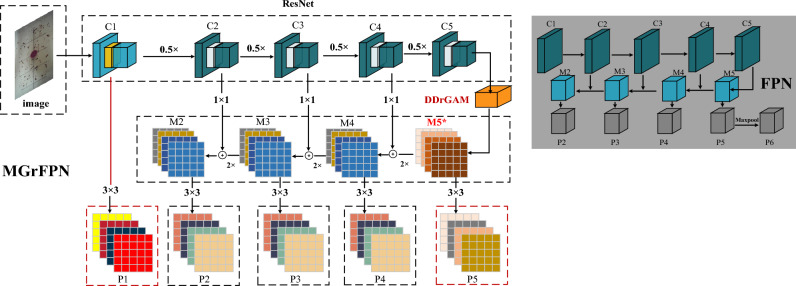
MGrFPN network structure. The left side of the figure shows the structure of MGrFPN, while the upper right part shows the structure of FPN.

Therefore, we have improved the output structure of the original FPN and designed a new feature pyramid structure-MGrFPN. As shown in Fig. 3, for the bottom-up path, according to the settings in FPN, the outputs of the last convolutional layers of the first to fifth convolutional blocks of Resnet50^27^ are denoted as {C1, C2, C3, C4, C5}; top-down paths and lateral connections are denoted as {P2, P3, P4, P5}, respectively.

To increase the amount of information about the tobacco beetle in the image, we add the feature maps of the C1 layer to the output of the traditional FPN and use enough shallow information to capture more detailed information about the small object and assist the precise location of the objects. The size of the tobacco beetles in the image is distributed between 30 px–80 px, and after 5 times downsampling, the feature maps only occupy about 0.94 px–2.5 px. Because the receptive field is too small, it is difficult to extract global semantic information. If the receptive field is too large, it will pay too much attention to the background information, which will bring a large amount of irrelevant noise, resulting in the loss of the features of the tobacco beetle itself. In response to this problem, we improve the traditional method: instead of using the P6 layer formed by downsampling the feature map of the P5 layer as an output branch of the FPN, we add a module DDrGAM to enhance the salient features in the P5 layer, after forming a new M5* feature, 3 × 3 convolutional is performed to obtain a new P5 layer features as the output of the FPN.

### Dual-path deformable receptive field guided attention module (DDrGAM)

**Figure 4:**
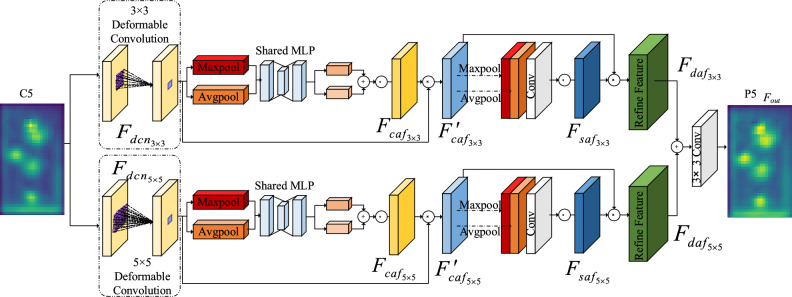
Deformable receptive field guided attention dual-path module (DDrGAM).

To further improve the detection accuracy, we design a DDrGAM module, which consists of two branches in parallel: a deformable convolutional^28^ and a feature attention block^29^ cascade. As shown in Fig. 4, The module uses the deformable convolutional kernel to extract the effective features, guides the feature attention block to focus on the effective features, and forms a mode of acquiring effective features-focusing on effective features-fusion effective features. Such a combination method can not only extract richer detailed information about tobacco beetles but also eliminate redundant features and fuse effective features.

Firstly, the module performs feature enhancement through deformable convolutional, that is, using deformable convolutional on the output of the feature extraction network to further obtain effective features.

Acquiring effective features stage: we denote the feature maps generated by C5 ∈ *R *^*H*×*W*×*C*^*.* Due to the small size of the tobacco beetle object, we use two different deformable convolutional kernels of 3 × 3/5 × 5 for feature enhancement to obtain the detailed features of the objects. As shown in formula (1).

1 $$F_{dcn} = Defconv(C5)$$

*F*_*dcn*_ represents the feature map after deformable convolutional. The convolutional kernel of deformable convolutional adds an offset at the position of each sampling point, which can concentrate more on the effective feature region. Moreover, by assigning different weights to the offset modified region, more obvious features are obtained. As shown in formula (2).

2 $$y(p) = \sum\limits_{k = 1}^{K} {w_{k} } \cdot x(p + p_{k} + \Delta p_{k} ) \cdot \Delta m_{k}$$

**Figure 5:**
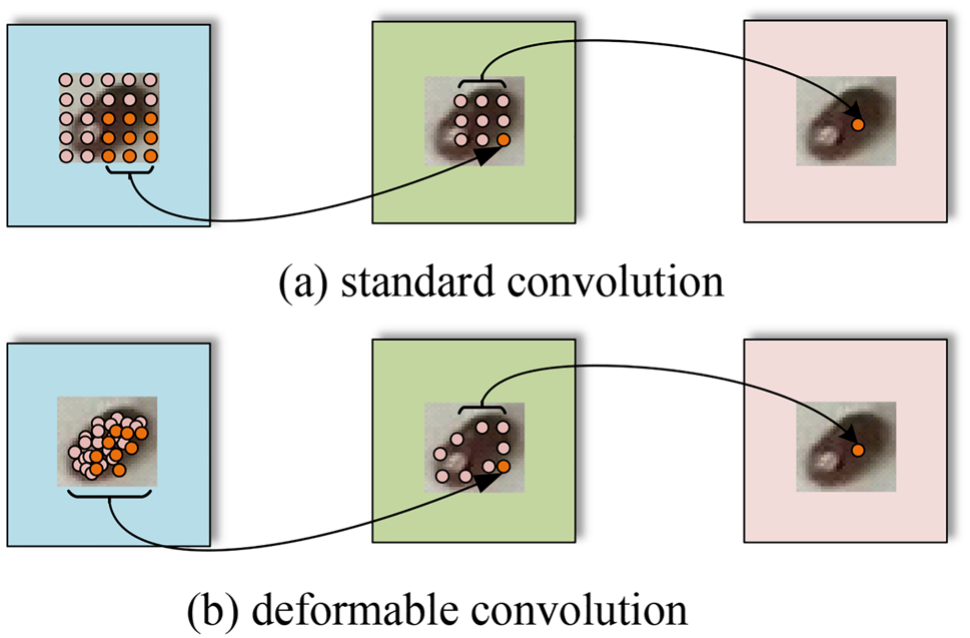
Deformable convolutional feature extraction process.

*k* denotes the sampling point, *x*(*p*), *y*(*p*) denotes the input feature map x*,* and output feature map *y* at the *p* position, respectively. *w*_*k*_, *p*_*k*_ denote the weight and predestined offset of the *k-th* position, and *K* denotes the size of the convolutional kernel at the sampling position. $\Delta {p}_{k}$ and $\Delta {m}_{k}$ are the learnable offset and modulation scalar at the *k-th* position, respectively. In this paper, the offset is used to find $\Delta {p}_{k}$ the regional position of the effective information of tobacco beetle, and the adjustment parameters $\Delta {m}_{k}$ are to give weight to the found position, which ensures the accurate extraction of effective information from these two aspects. Deformable convolutional is used to extract shape features of tobacco beetles, and the sampling area is no longer the original rectangle but changes according to the shape of the object. The shape of the tobacco beetle is an ellipse. By adding an offset to the sampling points on the input feature map, the original rectangular sampling area becomes similar to an ellipse, and a shape feature that is more in line with the object itself is obtained to achieve the effect of feature enhancement. Figure 5. illustrates the fixed receptive field in standard convolution (a) and the adaptive receptive field in deformable convolution (b), using three layers. (a) is a standard 3 × 3 square matrix sampling, and (b) is a non-standard shape sampling, but the sampling point is still 3 × 3. The activation is from a 3 × 3 filter. Top: Activation cells in tobacco beetles. Middle: To get the sampling process performed by the top activation unit. Bottom: 3 × 3 filter sampling area to get the middle layer. The highlighted positions correspond to the cells highlighted above. Deformable convolutional can sample more closely to the shape and size of objects, whereas standard convolutional cannot.

Focusing on effective features (semantic information and detail information): Since the deformable convolutional increases the receptive field, while enhancing the shape features of the tobacco beetle, it will generate some invalid background information. Therefore, it is necessary to compress the redundant information, reduce the noise, and focus on the features with a high correlation with the object. Therefore, we design a feature attention block connected after the deformable convolutional block, which combines channel attention^30^ and spatial attention^31^ mechanisms. Channel attention and spatial attention, respectively, contribute to features for object classification and localization. By paying attention to the semantic information and detailed information that need to be emphasized, irrelevant interference features such as the background can be eliminated.

We input the feature map *F*_*dcn*_ ∈ *R *^*H*×*W*×*C*^ generated by deformable convolution into the feature attention module to obtain the channel attention map *Fcaf*, which is used to assign the weights of each feature channel. *Fcaf* is a 1 × 1 × C channel attention map. The weight of each dimension on the map represents the importance and relevance of the feature layer corresponding to that dimension to key information. The larger the weight, the higher the correlation, which guides DDrGAM to pay attention to the channels with high correlation. Finally, after the weight of the feature channel is obtained, it is weighted to the original feature by multiplication, channel by channel, and the original feature re-calibration in the channel dimension is completed. The whole process can be described as(3):

3 $$F_{caf} = Sigmoid\left( {MLP\left( {Avgpool\left( {F_{dcn} } \right)} \right) + MLP\left( {Maxpool\left( {F_{dcn} } \right)} \right)} \right)$$

4 $$F^{\prime}_{caf} = F_{caf} \otimes F_{dcn}$$

Among them, ⊗ denotes the corresponding element multiplication operation. Channel attention can highlight interdependent feature maps and improve semantic-specific feature representation by mining the inter-dependencies between channel graphs.

To better characterize local regions of feature maps, we exploit the relationships between features to generate spatial attention maps. We send the feature map *F'*_*caf*_ refined by the channel attention map to the spatial attention module and obtain the spatial attention feature map *F*_*saf*_ of size H × W × 1, which encodes detailed information such as the color and texture of the object to which attention needs to be paid. At the same time, the noise is suppressed, and the important local information of the image is extracted. The spatial attention feature is described as:

5 $$F_{saf} = Sigmoid\left( {Conv\left( {Concat\left( {Avgpool\left( {F^{\prime}_{caf} } \right),Maxpool\left( {F^{\prime}_{caf} } \right)} \right)} \right)} \right)$$

Then the spatial attention feature map *F*_*saf*_ is multiplied by the refined feature map *F'*_*caf*_ of the channel attention map to obtain the feature block output *F*_*daf*_ after double attention adjustment, which is described as follows:

6 $${F}_{daf}={F}_{saf}\otimes \, {F}_{caf}{\prime}$$

Fusion effective features: To enhance the feature expression ability of the network and reduce missed detection, we propose a Dual-path Deformable receptive field Guided Attention Module method. Considering the small object size of tobacco beetles, 3 × 3 and 5 × 5 deformable convolutional kernels are used to cascade feature attention blocks to form two channels, respectively. Specifically, the information of the input feature map is augmented with a deformable convolutional structure at two scales. Then, the enhanced features are passed through the spatial attention module, which can more precisely focus on the contextual features, thereby extracting the location information of the two paths in each channel feature map. Then, the cross-dimensional interaction is constructed by extracting the channel attention weight of the feature map, the attention weight of the corresponding channel is recalibrated, and the object classification information of the two paths is obtained in the feature maps. Finally, the 3 × 3 convolutional kernel is used to integrate the output of the multi-scale feature by the two branches to eliminate the aliasing effect generated by the fusion of different receptive field feature maps in feature fusion, and obtain the output features *F*_*out*_ of the attention module guided by the dual-path deformable receptive field.

7 $$F_{out} = Conv_{3 \times 3} \left( {F_{{daf_{3 \times 3} }} + F_{{daf_{5 \times 5} }} } \right)$$

**Figure 6:**
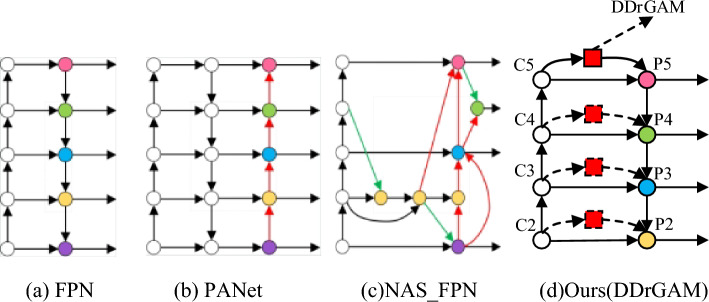
Feature pyramid network design.

The main optimization structures for feature pyramids are shown in Fig. 6a–c. DDrGAM proposed in this paper can be integrated into any layer connected by the backbone network and the feature pyramid network, as shown in Fig. 6d, which is a plug-and-play module, it can be directly applied to CNN structure. Through ablation experiments, it is found that adding the DDrGAM between C5 and P5 has the best effect, so other comparative experiments in this paper only add this module between C5 and P5. Compared with PANet^18^ and NAS_FPN^20^, this method does not add more fusion paths after the traditional prediction output but adds parallel feature information based on the feature extraction network, to perform feature enhancement and fusion. It can be seen that this method further enhances the feature expression ability without increasing the network complexity.

### Tobacco beetle monitoring system

Our method can be seamlessly integrated into existing tobacco beetle monitoring systems, achieving smooth integration. By efficiently packaging the developed MGrFPN-DDrGAM algorithm, we can build a powerful tobacco beetle detection software system. When combined with dedicated tobacco beetle image acquisition devices, this software system will form a comprehensive and efficient tobacco beetle detection system, providing strong support for practical applications. This integration not only enhances the overall performance of the system but also ensures its accuracy and reliability in real-time monitoring and analysis of tobacco beetle activities.

**Figure 7:**
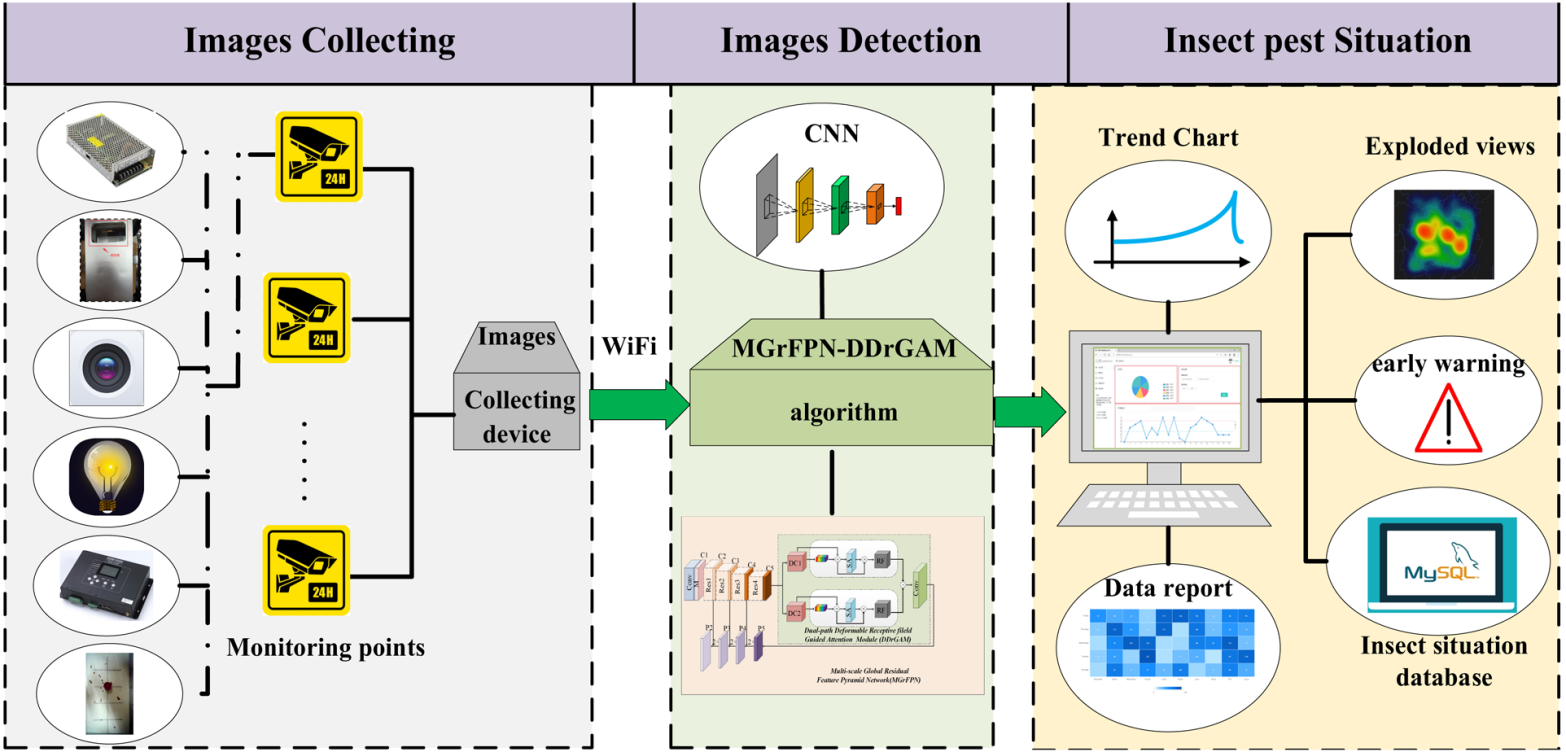
Schematic diagram of MGrFPN DDrGAM algorithm embedded in tobacco beetle detection system.

The tobacco beetle detection system based on convolutional neural networks consists of an image collection terminal, image processing algorithm, data analysis, and user display interface, as illustrated in Fig. 7. The image collection terminal includes an image sensor (camera), light source, controller, and dustproof trapping device, which are used for collecting images of pests. These images are then transmitted via network communication lines to the server for detection and identification. The image processing algorithm employs the MGrFPN-DDrGAM method for small object detection of pests. After analysis, the detection results are saved to the database and sent to the graphical user interface for visualization.

This system uploads the collected data for detection at intervals of one hour. The data display platform performs statistical and visual analysis of the information, enabling automatic statistics of the number of sawtooth beetles at each monitoring point. It generates data reports, explosion diagrams, and trend charts, as well as providing real-time alerts for the level of insect infestation, thus achieving intelligent monitoring of insect conditions.

During the deployment of the algorithm, it is advisable to first test the performance of the detection system on the PC end. Once it meets the established requirements, implementation can proceed on the embedded end, in conjunction with the pest image collection device, to form a complete pest detection system. By testing its effectiveness, reliability, and user experience within a local area, and ensuring that all metrics meet the established requirements, the system can then be promoted and applied more widely.

## Experiments and analysis

### Establishment of the dataset

Aiming at the task of tobacco beetle detection, we construct a dataset according to the real scene of a tobacco factory. A total of 28,080 images are collected, including whether the tobacco beetles are densely distributed, whether the pasteboard has shredded tobacco and whether the light is uniform, etc. The images include two kinds of environments: the real environment (taken from the smoke factory) and the simulated environment. Shooting with a white light camera, the size of the image is 1080 × 1920 pixels.

Real-world tobacco beetle data collection. According to the living habits of tobacco beetles and the historical record data of silk-making workshops, the dataset of the real environment mainly considers the influence of various environmental factors such as light intensity, temperature, humidity, dust concentration, etc. We captured tobacco beetle images in a variety of conditions.

Simulated environmental tobacco beetle data collection. To simulate the real environment of a tobacco factory, about 500 live adult tobacco beetles were manually placed on the trap pasteboard in the tobacco beetle image acquisition device in the laboratory for shooting.

**Figure 8:**
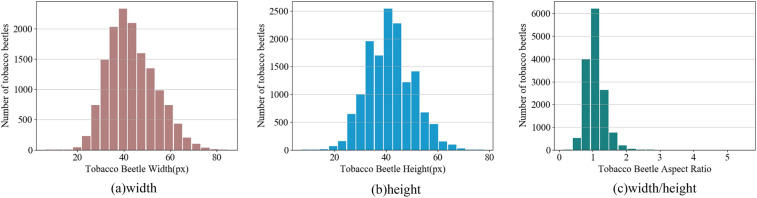
Statistical histogram of bbox width (w)/height (h)/aspect ratio (w/h) of tobacco beetle annotation boxes in all images in the self-built dataset.

The details of the tobacco beetle dataset are shown in Table 2 and Fig. 8. The width and height distribution of the tobacco beetle frame is approximately normal distribution, mainly concentrated between 30 and 50 px, of which 40 px is the most. The aspect ratio distribution of tobacco beetles is largely concentrated at 1.0, and the detection object is mainly 40 px × 40 px. This paper utilizes the Pascal VOC format and employs LabelImg as the annotation tool to create a custom dataset of small tobacco beetle objects.

**Table 2:** The distribution and complex background statistics of tobacco beetles in the data set.

Item	Sparse or dense		With or without cut tobacco			Light conditions	
	sparse	dense	Without	Little	Much	Even	Uneven
Number (pcs)	16,093	11,987	9971	8910	9199	16,509	11,571
Scale (%)	57.312	42.688	35.510	31.731	32.759	58.794	41.206

### Hyperparameter settings and training methods

All experiments in this paper are carried out in the hardware environment of the GPU model of NVIDIA GTX1080Ti, and the deep learning framework used is Pytorch1.2.0. There are a total of 28,080 images in the self-built dataset, which are used for training, validation, and testing in a ratio of 3:1:1, of which 16,848 images are used for training, 5616 images are used for validation, and 5616 images are used for the test. Images are resized to 1344 × 768 pixels according to the image aspect ratio. During the training process, the ImageNet pre-trained model is used as the initial weight of the backbone. The learning rate was initialized to 0.001, the batch size was set to 4, and a total of 70 epochs were trained. To fine-tune the model, experiments are set to reduce the learning rate to 1/3 of the original after training for 16 epochs and reduce it to 1/3 of the previous learning rate after training for 30 epochs. The Stochastic Gradient Descent (SGD) optimizer is applied to the network parameters, and default values are applied to other parameters.

To evaluate the detection performance, the experiments in this paper use common evaluation indicators in the object detection field, including Recall and Average-Precision (AP). The Recall is the proportion of all positive samples in the test set that are correctly identified as positive samples. Since this paper only involves one kind of object, Recall is the Accuracy, and AP is the Precision-Recall (PR) curve for the lower area of *P*(*R*), the calculation formulas of Recall and AP are shown in formula (8) and formula (9).

8 $$Recall = \frac{TP}{{TP + FN}}$$

9 $${\text{AP}} = \int_{0}^{1} {P(R)dR}$$

Formula (8), *TP* denotes the number of correctly identified examples of tobacco beetles, and *FN* denotes the number of incorrectly identified examples of tobacco beetles.

### Ablation experiments

The ablation experiments shown in Table 3 are designed to verify the effect of the DDrGAM. We denote the outputs of the last convolutional layers of the second to fifth convolutional blocks of Resnet50^27^ as {C2, C3, C4, C5}, DDrGAM is added between C2 and P3, C3 and P3, C4 and P4, C5 and P5, and all layers respectively to verify its function. The results show that the module works better at high levels. The main reasons are: (1) the object location of the lower layer is relatively accurate, but the noise is large. Since the size and color of the cut tobacco are similar to the tobacco beetle, which has a particularly large impact on the detection of tobacco beetles, so when the module is added to the bottom layer the performance is not very good; (2) The high-level feature semantic information is rich, but the object location is relatively rough. Therefore, we added the DDrGAM module at a high level, which can not only enhance the object features but also capture the regional location of the tobacco beetle.

**Table 3:** Ablation experiments on position setting of DDrGAM module.

Methods	Setting	Faster R-CNN^4^			Cascade R-CNN^32^		
		AP_0.5_	AP_0.6_	AP_0.7_	AP_0.5_	AP_0.6_	AP_0.7_
DDrGAM + FPN	@C2 ~ P2	89.9	87.3	59.0	87.5	74.2	48.0
	@C3 ~ P3	90.0	87.2	59.2	88.4	75.4	47.8
	@C4 ~ P4	90.1	87.1	58.8	87.8	74.8	48.4
	@C5 ~ P5	89.7	**88.1**	**61.3**	**89.8**	**87.7**	**60.4**
	@C2 ~ P2 @C3 ~ P3 @C4 ~ P4 @C5 ~ P5	**90.2**	87.4	57.7	88.9	76.5	48.8

Significant values are in bold.

For example, DDrGAM + FPN@C2 ~ P2 means adding DDrGAM between the C2 lay-er and P2 layer of FPN; DDrGAM + FPN@C2 ~ P2@C3 ~ P3@C4 ~ P4@C5 ~ P5 means adding DDrGAM between all the C2 layer and P2, C3 and P3, C4 and P4, C5 and P5 layers.

In the DDrGAM module, we conduct an in-depth experimental study on the selection of convolutional kernel sizes. To comprehensively assess the impact of different kernel sizes, we test with three common sizes: 3 × 3, 5 × 5, and 7 × 7. It is clearly observed from Table 4 that when using these three convolutional kernel sizes individually, the 3 × 3 and 5 × 5 kernels exhibit superior performance.This result indicates that when dealing with high-level features, due to multiple downsampling, the pixel ratio of small targets in the feature map is already very limited. In such cases, using large convolutional kernels makes it challenging to effectively capture the features of these small targets. In contrast, the combination of 3 × 3 and 5 × 5 convolutional kernels not only captures detailed features highly relevant to the targets in the feature map but also improves the learning of multi-scale features through attention-guided feature aggregation.

**Table 4:** Ablation experiments on deformable convolutional attention paths and Kernel size of DDrGAM Module.

Module	Path	Deformable convolutional size	Faster R-CNN^4^			Cascade R-CNN^32^		
			AP_0.5_	AP_0.6_	AP_0.7_	AP_0.5_	AP_0.6_	AP_0.7_
DDrGAM	1	3 × 3	88.2	87.3	60.9	89.0	86.8	59.4
	1	5 × 5	87.9	87.0	60.5	88.3	86.0	58.7
	1	7 × 7	85.4	85.1	57.9	87.1	84.6	56.4
	2	3 × 3, 5 × 5	**89.7**	**88.1**	**61.3**	**89.8**	**87.7**	**60.4**
	3	3 × 3, 5 × 5, 7 × 7	88.0	86.7	60.3	88.0	85.7	58.0

Significant values are in bold.

To assess the impact of DDrGAM and the multi-feature fusion approach in our framework, we conduct ablation experiments. A meticulous comparison is made between object detection results with and without the DDrGAM module. From Table 5, it is evident that the introduction of DDrGAM produces varying effects under different feature fusion methods. The improvement in AP0.5 is relatively modest, while AP0.6 and AP0.7 show substantial enhancements. This suggests that, in more complex detection tasks, the superiority of the DDrGAM module becomes more pronounced. This may be attributed to DDrGAM's ability to enhance contextual information while exhibiting advantages in handling localization tasks. Furthermore, the experimental results also indicate that adopting the {P1, P2, P3, P4, P5} multi-feature fusion approach confers significant advantages in detecting small targets. This is primarily because this fusion method effectively leverages lower-level information, enhancing the model's ability to capture details.

**Table 5:** Ablation experiments oni scale feature fusion method.

DDrGAM	Multiscale features	Faster R-CNN^4^			Cascade R-CNN^32^		
		AP_0.5_	AP_0.6_	AP_0.7_	AP_0.5_	AP_0.6_	AP_0.7_
	{P2, P3, P4, P5,P6}	89.5	85.0	55.3	88.4	75.4	49.1
√	{P2, P3, P4, P5,P6}	89.7	88.1	**61.3**	**89.8**	**87.7**	**60.4**
√	{P1,P2, P3, P4, P5}	**91.4**	**88.9**	**62.2**	**90.8**	**88.1**	**61.8**

Significant values are in bold.

### DDrGAM performance

Based on using Faster R-CNN^3^ and Cascade R-CNN^32^ as the detection network and, Resnet50^27^ as the backbone network, we compare methods whether embedding the DDrGAM in the FPN. As shown in Table 6, the proposed DDrGAM module achieves better performance. With the methods of embedding the FPN with the DDrGAM module, the detection precision is improved significantly. In the framework of the Faster R-CNN^3^ algorithm, when the IoU is 0.5, the AP is improved from 89.5 to 89.7% (+ 0.2), and when the IoU is 0.6, the AP is improved from 85.0 to 88.1% (+ 3.1), and when the IoU is 0.7, the AP is improved from 55.3 to 61.3% (+ 6.0). In the framework of the Cascade R-CNN^32^ algorithm, when the IoU is 0.5, the AP is improved from 88.4 to 89.8% (+ 1.4), and when the IoU is 0.6, the AP is improved from 75.4 to 87.7% (+ 2.3), and when the IoU is 0.7, the AP is improved from 49.1 to 60.4% (+ 11.3). The comparative experiments under the framework of both Faster R-CNN^3^ and Cascade R-CNN^32^ show that the proposed algorithm is an effective and certain generalization.

**Table 6:** Performance (%) for different methods.

Methods	IoU = 0.5		IoU = 0.6		IoU = 0.7	
	Recall_0.5_	AP_0.5_	Recall_0.6_	AP_0.6_	Recall_0.7_	AP_0.7_
Faster R-CNN + FPN	96.1	89.5	90.6	85.0	69.8	55.3
Faster R-CNN + DDrGAM + FPN	**97.4**	**89.7**	**93.4**	**88.1**	**77.2**	**61.3**
Cascade R-CNN + FPN	92.5	88.4	85.8	75.4	66.9	49.1
Cascade R-CNN + DDrGAM + FPN	**96.4**	**89.8**	**92.2**	**87.7**	**74.0**	**60.4**

Significant values are in bold.

**Figure 9:**
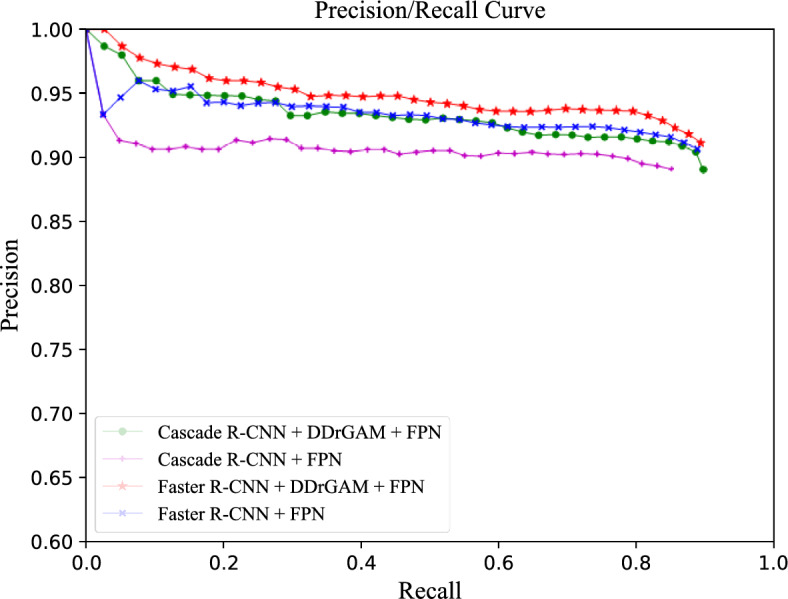
Precision-recall curve for DDrGAM performance.

We show the Precision/Recall curves of two state-of-the-art network models (Faster R-CNN^3^ and Cascade R-CNN^32^) after adding the DDrGAM module in Fig. 9. Obviously, higher Precision and Recall can be obtained after adding the DDrGAM module, and with the increase in Recall, the Precision can maintain a high value, which shows that our method can effectively reduce the false rate.

### MGrFPN–DDrGAM performance

To verify the performance of the network based on MGrFPN, the method of this paper is compared with the current mainstream detection network and feature pyramid network. The compared networks include Faster R-CNN^3^, SSD^9^, and YOLOv3^7^, and feature pyramid Networks include NAS_FPN^20^. In addition, it also includes CARAFE_FPN formed by embedding CARAFE^15^ into traditional FPN and the feature pyramid network of ASPP^16^ is fused to form ASPP_FPN. The experimental results are shown in Table 7. The Average Precision of the algorithm proposed in this paper is: 91.4% (IoU = 0.5), 88.9% (IoU = 0.6), 62.2% (IoU = 0.7). Moreover, the Average Precision and Recall of the algorithm designed in this paper are both the highest. When the IoU is 0.5 and 0.6, compared with CARAFE_FPN, the Average Precision is improved by 1.6%; when the IoU is 0.6 and 0.7, compared with ASPP_FPN, the Average Precision is improved by 2.0% and 3.5% respectively, our method is better than the current mainstream algorithm.

**Table 7:** Recall rates (%) and average precision (%) for different networks.

Methods	IoU = 0.5		IoU = 0.6		IoU = 0.7	
	Recall_0.5_	AP_0.5_	Recall_0.6_	AP_0.6_	Recall_0.7_	AP_0.7_
Faster R-CNN^3^	84.50	77.60	69.80	55.00	44.10	29.30
Faster R-CNN + FPN^13^	96.10	89.50	90.60	85.00	69.80	55.30
EfficientDet^33^	72.32	67.85	51.73	42.80	35.00	17.85
SSD^9^	72.34	68.45	52.17	43.26	35.17	19.17
YOLOv3^7^	77.53	70.36	56.48	47.67	37.23	25.48
YOLOv8l^34^	81.37	77.41	61.20	53.00	42.35	29.13
NAS_FPN^20^	95.0	89.50	89.40	77.0	70.20	49.80
CARAFE^15^_FPN	95.10	89.80	90.40	86.3	71.10	56.70
ASPP^16^_FPN	95.20	89.60	90.41	86.9	72.00	57.70
MGrFPN–DDrGAM	98.40 (+ 3.2)	91.40 (+ 1.6)	93.80 (+ 3.39)	88.90 (+ 2.0)	78.60 (+ 6.5)	62.20 (+ 3.5)

**Figure 10:**
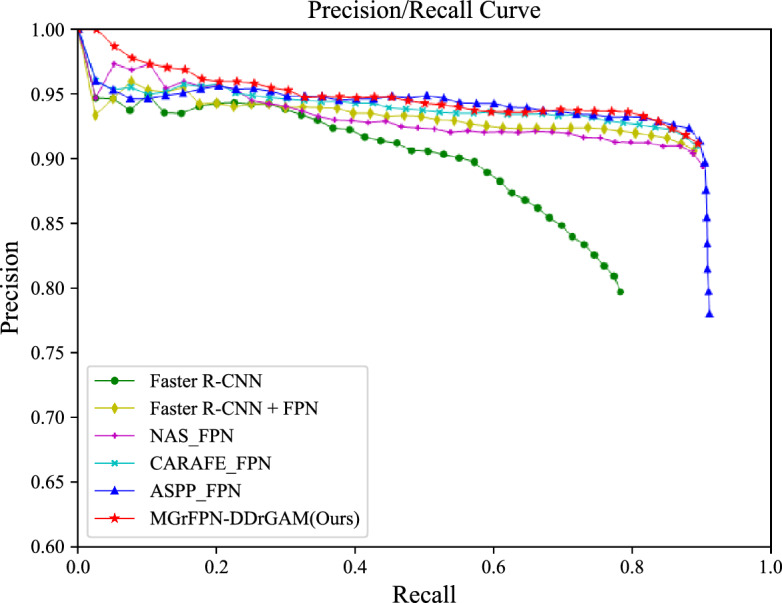
The precision-Recall curve for MGrFPN-DDrGAM performance.

Figure 10 shows the Precision/Recall curves of the detection performance of our proposed method and several other embedded feature pyramid network models. It can be seen from the figure that the detection performance based on the MGrFPN network is better than other methods. The classification and location accuracy of tobacco beetles have been greatly improved. There are two main reasons for this improvement: One is the design of the combination of two-path deformable convolution and attention in DDrGAM, which can meet the detection of scale-changing tobacco beetles. While learning the shape features of tobacco beetles, it can use attention to guide the fusion of position information, so that it can be easier to locate the object. The second is that the improved MGrFPN network increases the multi-scale features of small objects similar to tobacco beetles, reducing missed and false detection.

Although our method has demonstrated outstanding performance compared to mainstream algorithms, we have observed a phenomenon: there is a significant decrease in performance as the IoU increases. This phenomenon is not unique to our method but is commonly observed in various approaches. After thorough analysis, we believe there may be several reasons for this: Firstly, an increase in IoU implies increasingly precise localization of object bounding boxes. This not only requires our method to accurately detect the objects but also demands high precision in predicting the object bounding boxes. However, in practical applications, due to the diversity and complexity of objects in images, as well as background interference and other factors, accurately predicting object bounding boxes is a highly challenging task. Therefore, when the IoU threshold is raised, our method may face greater challenges, leading to performance degradation. Secondly, our method may have shortcomings in detecting occluded objects. These challenges may become more apparent as the IoU threshold increases, thereby affecting the overall performance of the method. To address these issues, we plan to take the following measures to improve our method in future work: Introduce more advanced feature extraction networks to enhance the representation capability of target features, thus more accurately predicting object bounding boxes. Adopt more refined object localization strategies, such as using methods like multiscale feature fusion or bounding box regression refinement, to improve the precision of predicting object bounding boxes. Strengthen the ability to handle small and occluded objects, for example, through improvements in loss functions, to better cope with these challenging scenarios.

To fully demonstrate the generalization performance of the proposed method in this paper and to conduct a fair comparison with existing methods, we choose to perform experimental validation on the COCO dataset. During this process, we will compare our results with the detection results of some existing methods on the COCO dataset to ensure the objectivity and reliability of the experimental results. The experimental data of these comparative methods are all from relevant literature.

The MS COCO dataset can be utilized for multiple tasks and features a rich diversity of data samples extracted from complex daily scenes. The dataset comprises 328,000 images and 2,500,000 labels, with over 1.5 million individual objects across a total of 91 object categories. Over half of the annotated images in the MS COCO dataset adhere to the definition of small objects.

Table 8 presents a comparison of the detection performance of mainstream small object detection methods on the COCO test-dev dataset with the method proposed in this paper. It can be observed that YOLOv5 achieves the highest scores in terms of AP. Compared to ResNet, YOLOv5 utilizes a more powerful backbone network, known as Modified CSP v5. However, its performance in terms of APs cannot surpass our method based on ResNet-101, MGrFPN-DDrGAM. Therefore, it can be inferred that even though the backbone networks of YOLOv4 and v5 possess higher feature extraction capabilities, our proposed method enhances the richness of features and the fusion of semantic information through design, thereby enhancing the ability to extract stronger semantic and contextual information. This further strengthens the feature representation capability, indicating that the improvements made by the proposed method are still relatively significant.

**Table 8:** Performance validation experiments on the COCO test-dev dataset with mainstream algorithms (%).

Method	Backbone	AP(%)	AP_50_	AP_75_	AP_S_	AP_M_	AP_L_
YOLOv3^7^	Darknet-53	42.4	63.0	47.4	25.5	45.7	52.3
EfficientDet^33^	Efficient-B0	33.8	52.2	35.8	12.0	38.3	51.2
PP-YOLOv2^35^	ResNet50-vd-dcn	49.5	68.2	54.4	30.7	52.9	61.2
YOLOv4^36^	CSPDarknet-53	43.5	65.7	47.3	26.7	46.7	53.3
YOLOv5^37^	Modified CSP v5	**50.4**	68.8	–	–	–	–
YOLOX^38^	Darknet-53	47.4	67.3	52.1	27.5	51.5	60.9
MGrFPN–DDrGAM	ResNet-50	48.9	69.2	53.3	30.7	51.4	59.1
MGrFPN–DDrGAM	ResNet-101	50.1	**69.7**	53.9	**31.5**	52.7	59.5

Significant values are in bold.

### Result visualization

**Figure 11:**
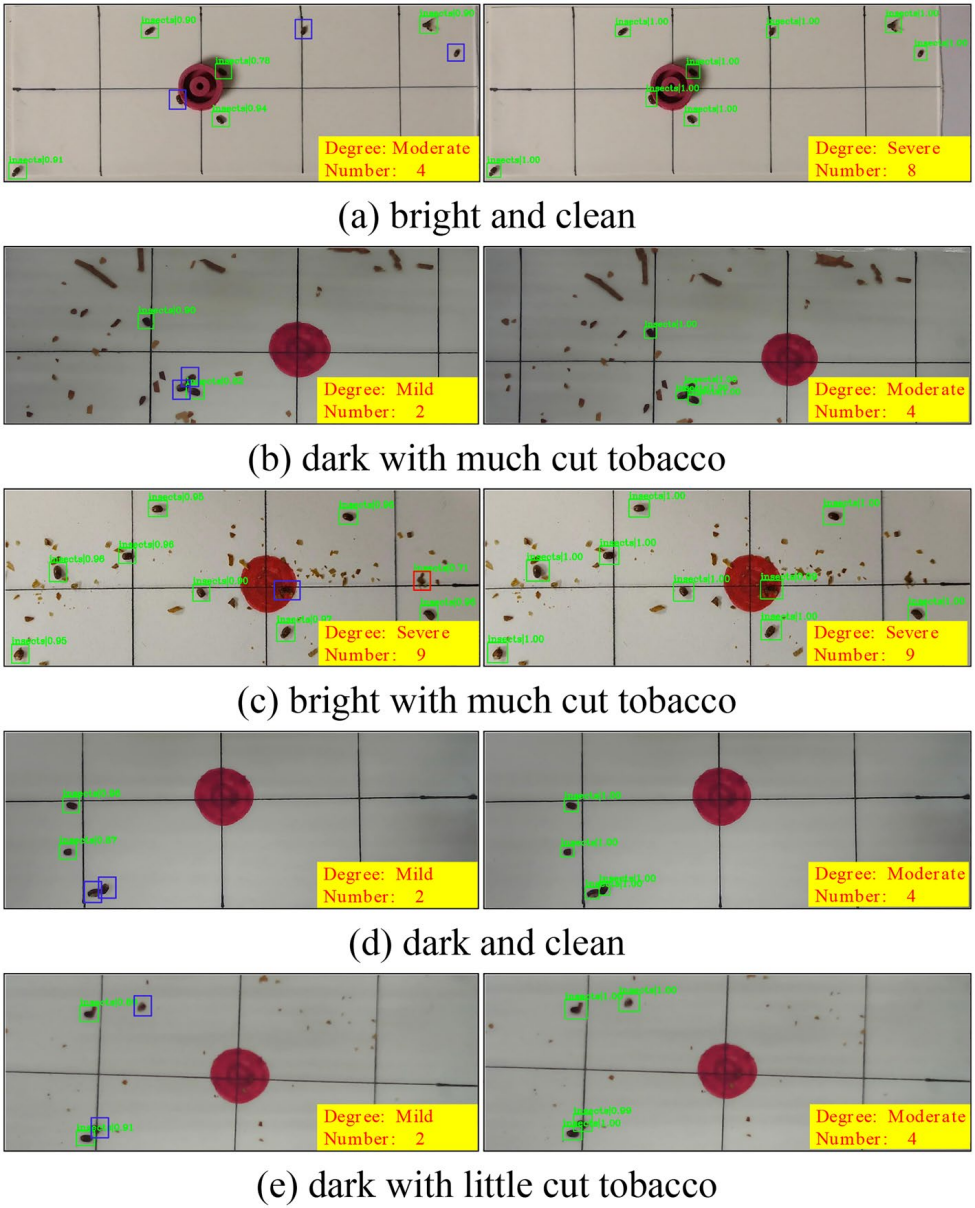
The detection results of different methods on the self-built dataset. A total of 5 scenes are included, and the green, red and blue rectangles represent true positives, false positives, and false negatives, respectively. The green number represents the confidence level.

Figure 11 shows the visualization results of five scenarios when the IoU is 0.6 under the Faster R-CNN^3^ framework. Each row shows the results of the base model (left) and our method (right). The experiment also shows the number of detected tobacco beetles and the degree of insect severity. The basic model has missed or false detection in various scenarios especially missed detection is very serious, which is very unfavorable for the control of tobacco beetles. Compared to the base model, our method can better detect small objects like the tobacco beetle with higher confidence and performs better when there is more noise (for example, tobacco leaves), darker light, and a higher IoU.

Our method exhibits robustness when facing challenges such as lighting variations, complex backgrounds, and beetle pose changes. Specifically:

Lighting variations: Regardless of whether the environment is bright or dark, our method accurately identifies and locates tobacco beetles, thus handling image inputs under different lighting conditions effectively.

Background complexity: Even in environments with much cut tobacco, our method can effectively extract beetle features, enabling precise detection and classification. Our model is capable of disregarding interference from the background and focusing on the beetle's characteristics.

Beetle pose changes: Our method maintains stable performance whether beetles are in clustered states or facing up, tilted sideways, or facing down. This is attributed to the model's robust adaptability to pose changes, allowing it to capture key features of beetles in different poses and achieve accurate recognition.

In summary, our method demonstrates good robustness in handling lighting variations, background complexity, and beetle pose changes, enhancing its versatility and reliability in practical applications.

## Conclusion

Aiming at the disadvantages of high time cost, low monitoring efficiency, poor real-time performance, and inaccurate data for manual insect statistics, we propose an automatic detection method for tobacco beetles as small objects in complex scenarios-MGrFPN-DDrGAM. The method uses a camera to capture insect images on the trap at a set acquisition frame rate, uses computer vision technology to perform intelligent object detection and recognition on tobacco beetle images, and automatically counts the number of tobacco beetles, which is a follow-up process for tobacco beetle control. Provide accurate and reliable insect monitoring data, and carry out insect warnings. In this paper, we build a multi-scene tobacco beetle dataset containing 28,080 images and use the Multi-scale Global residual Feature Pyramid Network (MGrFPN) for automatic extraction of multi-scale features and the Dual-path Deformable receptive field Guided Attention Module (DDrGAM) for effective feature automatic fusion to achieve the object classification, location and quantitative statistics of tobacco beetles. The MGrFPN network constructed in this paper can effectively fuse multi-scale features and enhance the expressiveness of features. At the same time, the DDrGAM module is designed. After the feature extraction network, deformable convolution is used to obtain effective features, which can better fit the shape and posture of tobacco beetles, reduce the interference of the background, and improve the classification performance of object detection. Then, the features are selectively fused through the feature attention block to enhance the generalization ability of semantic features and improve the location accuracy of object detection. at last. A large number of comparative experiments are carried out on the self-built dataset. Under different algorithm frameworks, the Average Precision of the method in this paper is superior to the compared advanced algorithms, which verifies the effectiveness of the method. The experimental results show that our method can recognize and accurately locate small objects, and can adapt to various morphological changes of small objects in complex environments and similar backgrounds, which is helpful for the precise detection of small objects such as tobacco beetles in complex environments.

Our method adopts advanced deep learning and computer vision techniques, which provide powerful feature extraction and classification capabilities. This enables our method to maintain stable performance in various complex and changing environments. Additionally, our method exhibits good scalability and customizability, allowing flexible adjustment and optimization according to specific application scenarios and requirements.

Although the method proposed in the paper demonstrates good performance in tobacco beetle detection, except for some issues discussed in the experimental section, there are still several limitations. Currently, our method relies primarily on a limited dataset for training and validation, which may restrict its ability to cover all potential tobacco beetle species and environmental conditions, thus affecting the model's performance in specific contexts. Additionally, despite achieving a balance between detection speed and accuracy, real-time application remains a challenge for devices with limited resources. Furthermore, although we validated the model's generalization ability on different datasets, in practical applications, the model may still face unknown challenges such as the emergence of new tobacco beetle species or environmental changes.

To overcome these limitations and further improve the method's performance, we plan to take the following measures in future research: Firstly, we will focus on expanding the dataset by collecting more types of tobacco beetle images and data under different environmental conditions to enhance the model's generality and robustness. Secondly, to reduce the demand for computational resources, we will explore model compression and acceleration techniques to enable real-time detection on resource-constrained devices. As new tobacco beetle species emerge and environmental changes occur, we will continuously update and optimize the model to ensure it maintains optimal performance. Additionally, we will consider integrating multimodal data such as sound and temperature sensor data to enhance the model's perceptual capabilities and further improve detection accuracy. Through these efforts, we hope to make greater contributions to the field of tobacco beetle monitoring.

## Data Availability

The datasets generated during and/or analysed during the current study are available from the corresponding author on reasonable request. Currently, our entire project is expected to conclude around June 2024. Upon the successful completion of the project, we will publicly release our complete code and dataset for other researchers to use, reference, and further expand upon. Before the project concludes, if any researchers are interested in our dataset and code and wish to gain access, we warmly welcome them to contact the corresponding author via email. We will strive to provide necessary assistance and support to meet their research needs.
